# Integrating Foundational and Clinical Science Remotely by Combining Team-Based Learning and Simulation

**DOI:** 10.1007/s40670-023-01817-9

**Published:** 2023-06-15

**Authors:** Joel Roberts, Qing Zhong, Rachel Linger

**Affiliations:** 1 Master of Science in Biomedical Sciences Program, Rocky Vista University, Englewood, CO 80112 USA; 2 Department of Biomedical Sciences, Rocky Vista University, Ivins, UT 84738 USA; 3 Department of Biomedical Sciences, Rocky Vista University, Englewood, CO 80112 USA

**Keywords:** Simulation, Team-based learning, Integration, Remote, Virtual, Inter-disciplinary, Health professions education

## Abstract

Integrating foundational and clinical science in medical and other professional healthcare degree programs has been well established as a means to enhance learning. However, implementation remains challenging, and a significant gap exists in guidance for non-professional degree programs to effectively accomplish both types of integration. Additionally, many modalities described in the literature are resource-intensive, scale poorly to larger groups, and are widely inaccessible. We present an online modality combining team-based learning and a simulation-based learning experience that fosters vertical and horizontal integration of physiology, pharmacology, and clinical science. The tools utilized include a vital sign simulator, video conferencing software, and a document-sharing platform. The activity demonstrated improved knowledge comparing pre- and posttests and evidence that the activity helped students integrate physiology, pharmacology, and clinical medicine. The novel structure is effective and accessible, uses open-source software and standard equipment available to most undergraduate and graduate faculty, and is adaptable to in-person, hybrid-remote, and fully remote delivery.

**Supplementary Information:**

The online version contains supplementary material available at 10.1007/s40670-023-01817-9.

## Introduction

Modern medical education has wrestled with the optimal integration of foundational and clinical sciences since the Flexner Report [1, 2]. The benefits of integration are well-recognized, but guidance for implementation is limited [3, 4]. Two main types of integration are described in the literature: vertical and horizontal [5, 6]. Both are believed to contribute to effective medical learning [3]. Vertical integration garners most of the focus in curricular design and research, connecting foundational sciences traditionally in the preclinical or pre-clerkship phase (“lower years”) of healthcare education to the clinical sciences traditionally in the clinical or clerkship phase (“upper years”) [6]. Horizontal integration connects concepts being emphasized at the same level of education and is often assumed to be implicit whenever vertical integration is used [6]. As examples of each, discussing how binding of a drug to specific receptors effectively treats a disease would be vertical integration (foundational pharmacology with clinical medicine), while describing the effect of those drug-bound receptors on organ function would be horizontal integration (foundational pharmacology with foundational physiology). Two methodologies that facilitate vertical and horizontal integration are team-based learning (TBL) and simulation [7].

TBL began formally in the 1970s, but became popular after publication of a TBL reference book by Michaelsen, Knight, and Fink in 2002 [8]. TBL has learners work in teams with a well-defined format that includes preparation, readiness assurance, and application exercises and, while traditionally in person, has been adapted to virtual team environments [8, 9]. It is an effective modality for teaching in healthcare education, improving test scores and being well-received by students [10–13].

Publications on using simulation in healthcare education in both clinical and foundational sciences began increasing in the early 2000s [3, 14, 15]. A good working definition of simulation is “a technique-not a technology-to replace or amplify real experiences with guided experiences that evoke or replicate substantial aspects of the real world in a fully interactive manner” [16, 17]. A simulation-based learning experience (SBLE) is defined as “an array of structured activities that represent actual or potential situations in education and practice” [17]. SBLE lends itself well to vertical integration, and, similar to TBL, is established as effective in healthcare education for improving test scores and being well-received by students [2–4, 18–21]. Minimal research exists on SBLE in non-clinical degree programs where foundational science content interacts significantly with clinical sciences, with only one paper identified with outcomes revealing the activity was well-received by students [22].

There is growing support for using TBL and SBLE together to teach pharmacology and other topics in healthcare education, although what simulations are used and how they are integrated or paired with TBL varies [23–29]. Many of these studies demonstrate that students enjoy TBL and SBLE when used together and some studies show quantitative improvements in learning with this methodology. Three information gaps were identified in the literature. First, very little data exist exploring student perceptions of integration with different learning modalities [26, 30]. Second, practices for using TBL and SBLE together are not well established (i.e., which simulation modalities to use, whether they are used in TBL prework, application activities, or paired sequentially, etc.). Third, how this methodology applies to pre-medical foundational science learners remains unknown.

A number of barriers exist to implementing and investigating the exciting, effective, and growing modality of combined TBL and SBLE. TBL requires a larger space than traditional lecture, as students must form groups that foster effective communication. Physical attendance poses an additional logistical challenge, especially to SBLE, and was particularly challenging during formal quarantine in the COVID era. SBLE suffers from physical space challenges as well, as simulations often are done with physical manikins, task trainers, standardized patients, and virtual reality equipment [18]. SBLE poses additional challenges, such as high cost, increased faculty/technician workload to run numerous simulations, cumbersome technology, and simulation equipment rarely accessible to non-clinical programs [18, 20, 30]. This study sought to address these challenges by designing a modified-TBL and SBLE event that was interactive, remote-compatible, inexpensive, effective for learning, and emphasized both vertical and horizontal integration. Successfully capturing these goals could make this approach one of the most widely accessible options for faculty interested in using TBL and SBLE in physiology, pharmacology, and clinical education in a pre-graduate-health Master of Science in Biomedical Sciences (MSBS) program.

## Materials and Methods

This research is a retrospective analysis of an educational activity that occurred during the Fall 2021 Physiology I course in the MSBS program at Rocky Vista University (RVU). The Rocky Vista University Institutional Review Board (IRB) approved this research project (“Vital Sign Simulator: Cause and Effect,” RVU IRB# 2022–027) and considered it exempt.

### Students and Faculty

Seventy-eight (78) students were enrolled in the RVU Master of Biomedical Sciences (MSBS) Program Physiology I course for the Fall 2021 semester. Students were located on two geographically distant campuses, with 43 students (55%) in Englewood, CO, and 35 students (45%) in Ivins, UT. The course director, an assistant professor of physiology with an MD degree, was located in Englewood, CO. Two additional faculty participated in the activities, one on each campus. Both are associate professors of pharmacology, one with a PhD degree and the other with MD and PhD degrees.

### Technology

The interactive class session was conducted virtually (Zoom Video Communications) using an online TBL platform (InteDashboard). Virtual patient scenarios were simulated using real-time vital sign display (Vital Sign Simulator, released under GNU General Public License, created by Florian Schwander) including adjustable heart rate, blood pressure, spO2, etCO2, respiration, and moving ECG. Pre- and posttest assessments and surveys were administered via Qualtrics.

### The Activity

The educational activity followed a modified TBL-format with use of an embedded SBLE. The self-directed preparation assignment was a table of pharmacologic drugs, their mechanisms of action, clinical uses, and physiologic effects. For each drug, either the mechanism of action or the physiologic effects were provided. Students were instructed to complete the table by choosing the most appropriate mechanism of action or physiologic effects from a list of options. Students were encouraged to work in teams to complete the assignment. The completed table with correct answers was provided before the live class session.

Six days after the self-directed preparatory assignment, students and faculty met virtually for a 2-h interactive class session entitled “Vital Sign Simulator: Cause and Effect.” The interactive class session continued to follow a modified-TBL format utilizing an online platform for TBL (InteDashboard). At the start of class, students were provided a weblink to an optional five-question individual assessment of their knowledge of autonomic physiology and pharmacology administered via Qualtrics. Next, the self-directed preparatory assignment was reviewed and the structure of the session was described. For the remainder of the class session, students worked in teams to review two virtual patient cases and work through guided cause and effect exercises.

**Fig. 1:**
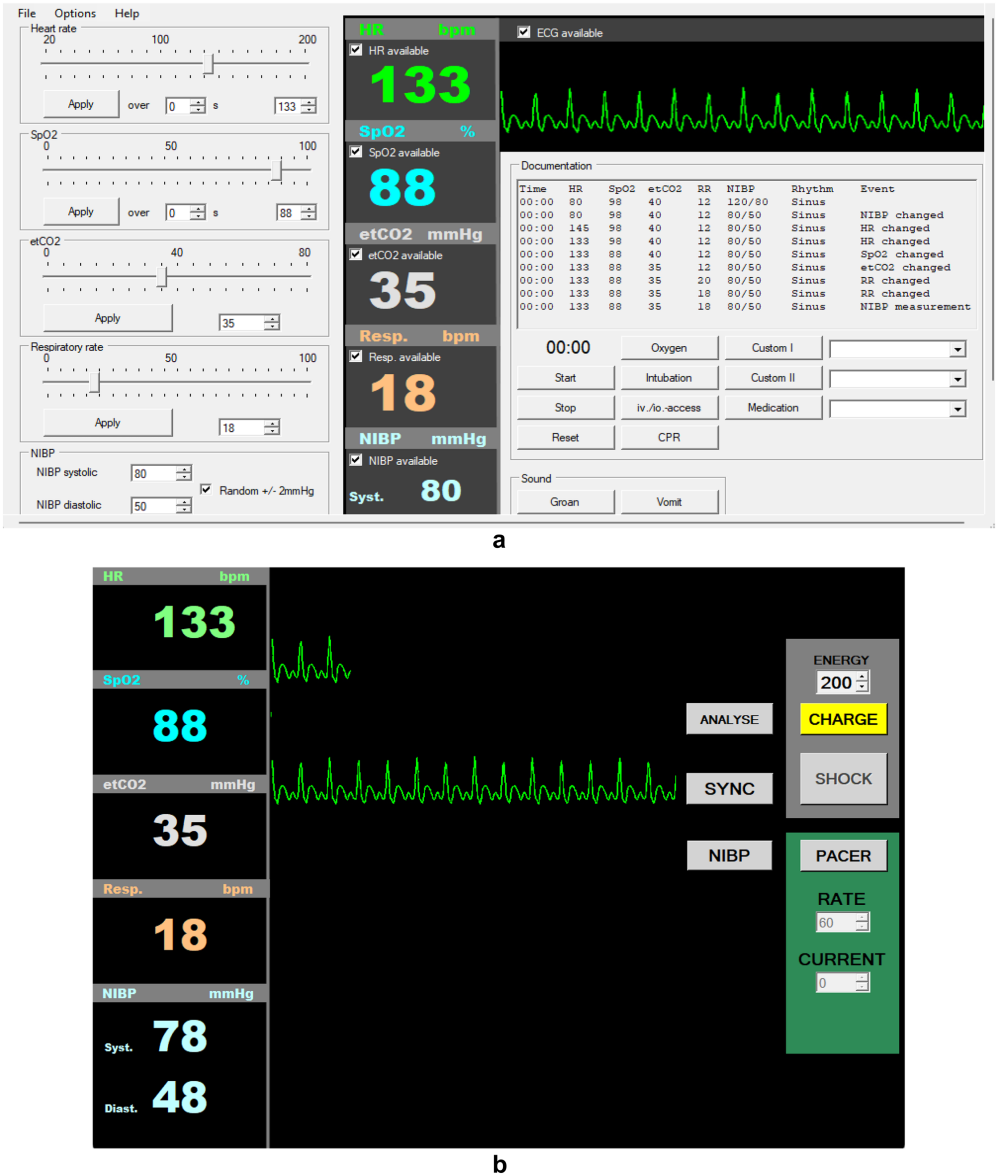
**a** Example screenshot illustrating the instructor manipulation panel of the vital sign simulator software. Instructors can control the availability and value of vital signs, display ECG, and patient sounds. **b** Example screenshot illustrating the read-only student view panel of the vital sign simulator software

Students received a written patient case scenario in the ZOOM chat including age, height, weight, gender, chief complaints, past medical history, surgical history, and social history. Patient scenarios were simulated using a real-time Vital Sign Simulator software, with modifiable vital signs including adjustable heart rate, blood pressure, spO2, etCO2, respiration, and moving ECG. The program includes an instructor manipulation panel (Fig. 1a) and a read-only student view panel (Fig. 1b). The student view panel was shared via ZOOM. Students were sent to ZOOM breakout rooms to complete team-based activities on the InteDashboard platform, with five to seven students per room. The primary objective during this phase was for student teams to develop a differential diagnosis and choose an information gathering strategy. Upon returning to the main ZOOM room, the virtual vital signs were changed and new physical exam findings and laboratory results were revealed in the ZOOM chat. Students and faculty discussed possible differential diagnoses and information gathering. Students then returned to ZOOM breakout rooms for another series of team-based activities that instructed students to refine the differential diagnosis, choose interventions, and explain physical signs. Back in the main ZOOM room, students and faculty discussed the patient case and students again observed cause and effect in real time as virtual vital signs, physical exam, and laboratory results were updated in response to student-selected interventions. For example, student-instructed administration of norepinephrine would result in elevated blood pressure and reflex bradycardia. A final debrief of the case revealed the intended diagnosis and most appropriate interventions.

The process was repeated with a second virtual patient case. After completion of both virtual patient cases, students were provided a weblink to an optional five-question individual assessment of their knowledge of autonomic physiology and pharmacology as well as nine (9) Likert scale items and four (4) open-ended questions to assess attitudes and perception of the educational activities. The questions assessing knowledge were identical on the pretest and posttest (the questions are provided as “Supplementary Materials”). Data was collected from anonymous survey responses housed in Qualtrics.

### Statistical Analysis

The difference between average scores on the pretest and posttest was analyzed using an unpaired *t*-test, but the normality test failed. A Mann–Whitney rank-sum test of the median scores was conducted with *p* < 0.05 and was significant. Likert scale responses to post-activity survey questions were enumerated as follows: strongly agree = 5 points, agree = 4 points, neutral = 3 points, disagree = 2 points, and strongly disagree = 1 points. The average score and median scores for Likert scale responses were calculated. Qualitative data from the four open-ended questions was not formally analyzed, although themes were informally identified on review of the responses.

## Results

### Evidence of Effectiveness from Enhanced Performance on the Posttest

**Fig. 2:**
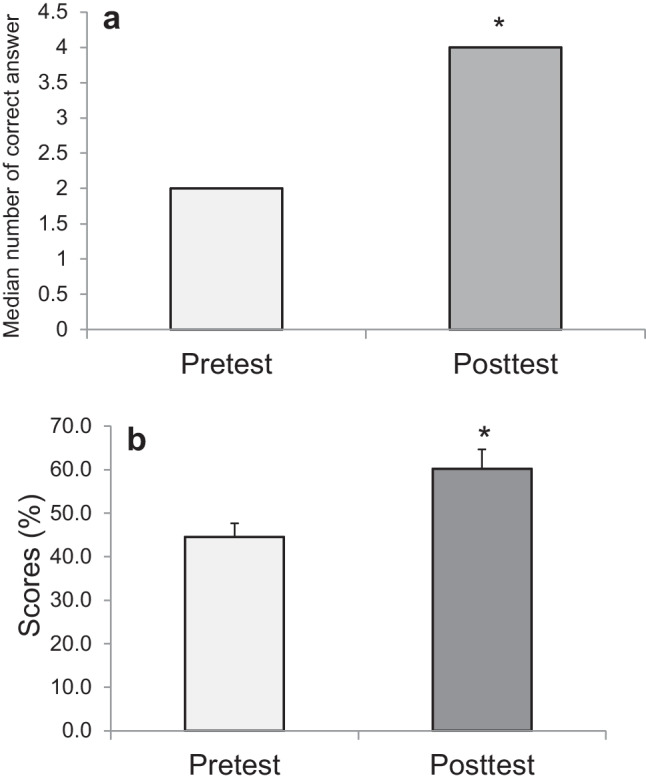
**a** The median number of correct answers for the five questions in pretest and posttest (pretest, *N* = 71; posttest *N* = 42; *p < 0.001). **b** Comparison between pretest scores and posttest scores (mean ± SE, pretest *N* = 71, posttest *N* = 42; *p < 0.001)

Out of 78 students enrolled in the Physiology I course, 71 students (91%) completed the pretest knowledge assessment and 42 students (53.8%) completed the posttest. As shown in Fig. 2, the median number of correct answers and average score for the posttest were significantly higher than those of the pretest (Fig. 2a: median number of correct answers: 2 vs 4, *p* < 0.001; Fig. 2b: mean ± SE, 44.6 ± 3.1 vs 59.6 ± 4.4, *p* < 0.001). On average, students scored 15% higher on the posttest compared to the pretest.

### Evidence of Effectiveness from Responses to Post-activity Survey

**Fig. 3:**
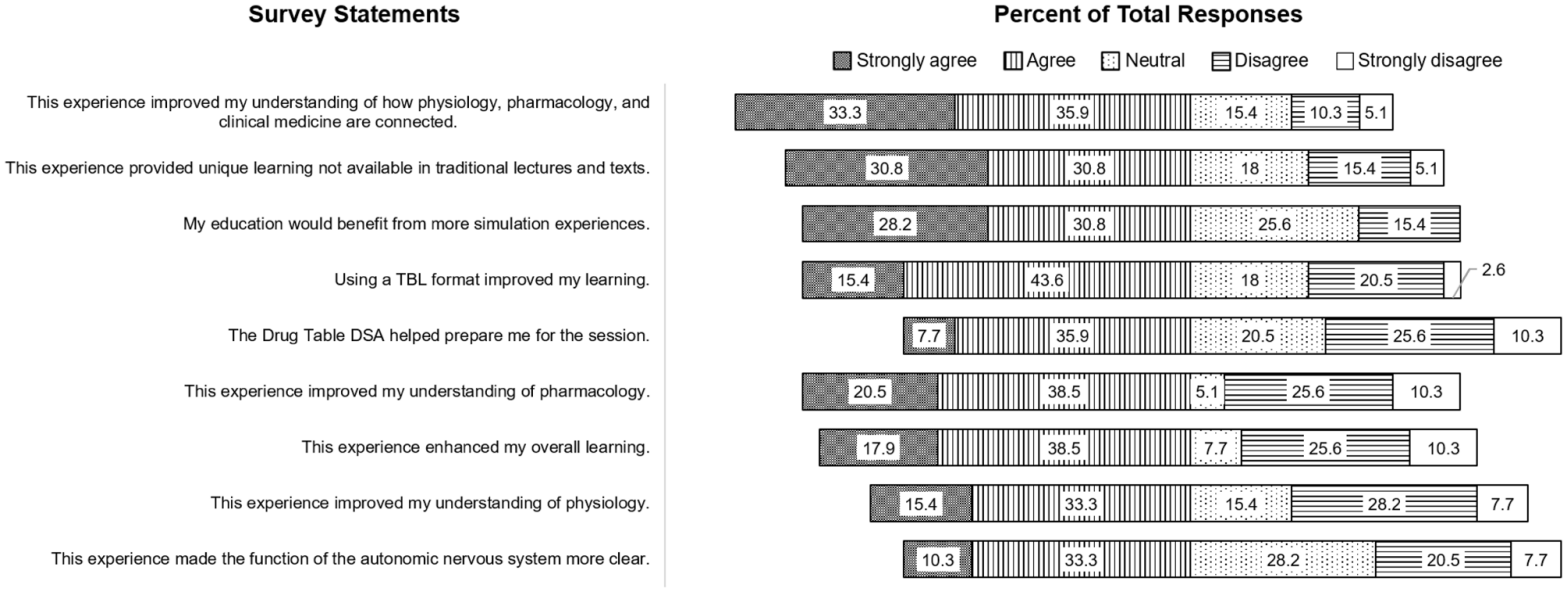
Visual representation of percent total responses to each survey statement within each category

The post-activity survey was completed by 39 (50%) students. Survey respondents made a value judgment of nine statements in relation to the aims and objectives of the vital-sign-simulation session. The Likert data are presented visually in Fig. 3. Detailed responses are summarized in Table 1. The statement that received the highest average rating described the vertical and horizontal integration of physiology, pharmacology, and clinical medicine, followed by statements that described the unique learning that is not available in traditional didactic lectures, benefit from more simulation experiences, and TBL-improved learning. Interestingly, learning individual disciplines (physiology or pharmacology) were rated lower than the integration aspect of the simulation session. The majority (69.2%) of students agreed or strongly agreed that “This experience improved my understanding of how physiology, pharmacology, and clinical medicine are connected,” while only 48.7% of students agreed or strongly agreed that “This experience improved my understanding of physiology” and 59% of students agreed or strongly agreed that “This experience improved my understanding of pharmacology.”

**Table 1:** Quantitative summary of student responses to the post-activity survey questionnaire (*N* = 39). In order to calculate median rating, mean rating, and standard error (SE), Likert scale responses were assigned numerical values as follows: strongly agree = 5, agree = 4, neutral = 3, disagree = 2, strongly disagree = 1

Questionnaire statement		Number and (percent) of total responses					Median rating	Mean rating	SE
		**Strongly agree**	**Agree**	**Neutral**	**Disagree**	**Strongly disagree**			
1	This experience improved my understanding of how physiology, pharmacology, and clinical medicine are connected	13 (33.3%)	14 (35.9%)	6 (15.4%)	4 (10.3%)	2 (5.1%)	4	3.8	0.2
2	This experience provided unique learning not available in traditional lectures and texts	12 (30.8%)	12 (30.8%)	7 (18%)	6 (15.4)	2 (5.1%)	4	3.7	0.2
3	My education would benefit from more simulation experiences	11 (28.2%)	12 (30.8%)	10 (25.6%)	6 (15.4%)	0 (0%)	4	3.7	0.2
4	Using a TBL format improved my learning	6 (15.4%)	17 (43.6%)	7 (18%)	8 (20.5%)	1 (2.6%)	4	3.5	0.2
5	The Drug Table DSA helped prepare me for the session	3 (7.7%)	14 (35.9%)	8 (20.5%)	10 (25.6%)	4 (10.3%)	3	3.1	0.2
6	This experience improved my understanding of pharmacology	8 (20.5%)	15 (38.5%)	2 (5.1%)	10 (25.6%)	4 (10.3%)	4	3.3	0.2
7	This experience enhanced my overall learning	7 (17.9%)	15 (38.5%)	3 (7.7%)	10 (25.6%)	4 (10.3%)	4	3.3	0.2
8	This experience improved my understanding of physiology	6 (15.4%)	13 (33.3%)	6 (15.4%)	11 (28.2%)	3 (7.7%)	3	3.2	0.2
9	This experience made the function of the autonomic nervous system more clear	4 (10.3%)	13 (33.3%)	11 (28.2%)	8 (20.5%)	3 (7.7%)	3	3.2	0.2

Four open-ended questions solicited free responses regarding student perceptions of the drug table assignment and the vital-sign simulation activity. The four questions were “What did you like about the DSA Drug Table assignment?”, “What would you change about the DSA Drug Table assignment?”, “What did you like about the simulation-based learning experience?”, and “What would you change about the simulation-based learning experience?”.

In summary of the qualitative data for “what did you like about the DSA Drug Table assignment” question, nearly all students (27/30, 90%) were able to provide a positive response (see Table 2). In summary of the qualitative data for “what did you like about the simulation-based learning experience” question, many respondents (18/30, 60%) liked the real-time vital sign changes upon administration of different drugs and mentioned the ability to integrate physiology, pharmacology to clinical medicine. While not directly solicited in these questions, the TBL approach was noted to enhance learning and discussion with peers (5/30, 16.7%). A small number of students (2/30, 6.7%) valued learning from three different faculty members with complementary expertise. Some examples of students’ positive responses to both open-ended questions are shown in Table 2 categorized by these four identified themes.

**Table 2:** Samples of student positive responses to open-ended questions about the self-directed drug table assigned as prework and about the live TBL-SBLE (vital-sign simulation) class session

**Positive themes**	**Student comments**
**Drug table assignment**	I loved the assignment, I thought it was a good way to learn about the different drugs and mechanisms and being able to discuss with a group about potential effects and clinical uses (student A)
	I enjoyed the experience of working through the mechanisms of action and physiological effects related to the given information (student B)
	It had the information already there; it was like putting together a puzzle without having to make the pieces first (student C)
**Real-time vital sign change and integration**	It was a hands-on way to see how medications affect physiologic states and I could connect more to it as it felt like we needed to decide in real-time with variables continuing to change (student D)
	I enjoyed being pushed to think about how all of what we have been learning is connected to “real-time” simulation of events in patients with disease processes. It helped make some strong connections to the material and how to apply these concepts to disease states (student E)
	It was nice seeing how different medications affected vital signs in real-time (student F)
**Team-based learning**	I liked discussing things in a group setting and being able to utilize my team’s experience and knowledge to learn more about a drug, and absolutely loved how we could see the vital sign changes (student G)
	Hearing how other people thinking helped me think of things I overlooked or did not put too much emphasis on (student H)
**Learning from three faculty members**	I liked that different professors were brought along to share their expertise (student I)
	I like the discussion with three faculty members (student J)

The qualitative data for the two “what would you change” questions revealed some useful suggestions for improving these learning activities in the future, which are summarized in Table 3. For example, some respondents requested that the session be scheduled at a time more distant from exams. Some students suggested increasing the duration of the live TBL-SBLE activity. Technology issues were cited by a few respondents including delayed information display in the chat due to suboptimal internet connectivity and distraction from the ZOOM chat. A few students would have preferred to meet in-person or in an in-person small group setting rather than holding class virtually on ZOOM. Some samples of students’ responses and potential solutions are summarized in Table 3.

**Table 3:** Sample student suggestions for improvement of the learning activities

**Student comments**	**Potential solutions**
I think the timing of the release of the drug table was challenging being a week prior to the block exam. (student K) Please give us more time to prepare for these sessions prior to class. It was overwhelming to get the case studies a few hours prior to the start of the simulation. (student L)	Provide the drug table assignment earlier so students can work on it as didactic sessions are presented
Sim’s are really cool but wasn’t a learning experience for me, overwhelmed with speed and all the drugs (student M)	Reduce the pace of the activity by either extending the duration or limiting the number of cases to one
I would like this to not be done right before the exam. Especially for those who are struggling and need the extra class time to study (student N) I found it stressful and confusing to have days before the block exam. (student O)	Schedule the session for a class session more distant from the exam
I would have liked to have small teams work together with the simulator ourselves. We would administer the drugs and see the effects after making a diagnosis. The simulation today felt very disjointed with responses from UT and CO (student P)	Revise the activity to include student operation of the vital sign simulator
I think breaking up into smaller groups with a professor would be easier to implement the TBL; over zoom was difficult to keep up and I felt I was unable to retain most of the information. I truly felt overwhelmed throughout the zoom. (student Q) I would change how its [sic] done on zoom and the information available. The internet connection trying to do it in a group was very unstable, and on zoom with many people talking over at once and too much going on in the chat it was difficult to keep up with what was being discussed. (student R)	Conduct the live TBL-SBLE session in-person rather than on ZOOM to avoid internet connectivity challenges

## Discussion

The most objective measurement of student learning in this experiment is the improvement in the multiple-choice question posttest section. The pretest was taken after independently completing the drug table, so improvements in the posttest represent additional learning attributable to the interactive TBL-SBLE session. The questions were written to directly measure ability to recall information and most closely approximate standardized exams used in most healthcare fields. The post-activity questionnaire is a subjective measure of student perceptions. Average scores and frequencies of all Likert scale responses show positive sentiments. Student perceptions of learning were positive overall, but not as high as student perceptions described in some of the literature with TBL or SBLE [31–34]. However, inverse correlation between student perceptions of learning and actual learning is described in the literature and use of data-supported modalities and techniques, such as spaced recall, interleaving, and active learning, should still be encouraged [31, 35, 36]. Of interest, the highest rated Likert scale item (statement 1) asked students about the connectedness of physiology, pharmacology, and clinical medicine (horizontal and vertical integration) in these learning activities. This statement was rated higher than distinct learning of pharmacology (statement 6) or physiology (statement 8). This unique early evidence suggests that students perceive integration and connectedness through a simulation-based interactive class session more strongly than they perceive learning the individual discipline content, although further investigation is warranted.

The unique design of this educational activity addresses many of the abovementioned significant barriers to use of TBL and SBLE. One barrier is space. TBL and SBLE often require a larger and more specialized physical space than traditional lecture. In the authors’ experience, simulation center access and simulation equipment are often limited and the tiered layout of traditional lecture halls makes the group work of TBL cumbersome. The modality described herein can easily be run in any single, large room with a projector, but can also be done hybrid-remote or completely remote with any video-conferencing software and a shared document platform. A hybrid-remote model can be achieved by groups gathering in person and then signing in to the remote session. If completely remote operation is desired, as described in this study, team members can be put into virtual breakout rooms for TBL application exercises and brought together as a class for report-out and discussion. SBLE poses additional challenges, such as high cost, faculty/technicians to run numerous simulations, cumbersome technology, and simulation equipment [18, 20, 30]. Our modality is inexpensive. Since the recent COVID pandemic necessitated virtual learning around the globe, many faculty and learners already have access to video conferencing software. While software specifically designed for TBL (e.g., InteDashboard) can make collaboration and report-outs simpler, these elements can be accomplished using any shared document platform (e.g., Google Docs, OneDrive). The vital sign simulator used is open-source software and is free. The software is intuitive, with an easy learning curve for the simulation technician (or session facilitator) and learners. Running the simulation for the entire session requires one person to serve as the simulation technician with either moderate clinical experience or a thorough “if–then” outline and only needs one faculty to manage report-outs, discussion, and technological details. The authors believe that the power of using TBL and SBLE is worth overcoming these barriers and that the methodology described in this study successfully address them by designing a combined TBL-SBLE event that was remote-compatible and required minimal resources while remaining interactive and effective. This approach may represent one of the most widely accessible options for faculty interested in using TBL and SBLE in clinical or foundational sciences.

To our knowledge, this is the first report of a vital sign simulation involving physiology, pharmacology, and clinical medicine for preclinical master program students. It is a virtual session fitting for remote, in-person, and hybrid learning, and cheaper than other types of simulators. It is an early address of the three abovementioned information gaps that were identified in the literature. First, this can provide a baseline of students’ perceptions of integration, which future modalities can be compared against. It also suggests a possible disconnect between perception of integration and perception of learning the individual content areas being integrated, as mentioned above, warranting further exploration. Second, we show that using a TBL format with SBLE during the application exercise component can be effective for learning. Third, this methodology is effective for pre-medical foundational science learners and use of TBL, SBLE, and their combination is worth investigating in other non-clinical degree programs.

There are some limitations to this study, areas for improvement of the modality, and areas for future research that warrant consideration. Our study design lacked a control group. While this allowed all learners enrolled in the class to have a comparable experience, it lacked comparison to a more standard class activity and limits our ability to compare effectiveness of different methods. While inexpensive and easy to execute, it was more resource-intensive than, for example, a traditional lecture. A similar limitation is the measure for learning. While pre-post knowledge tests and student perceptions are often employed to demonstrate that teaching methods were effective for learning, use of internal or external benchmarks could provide more validity. Post-activity survey response rate was low despite time built into the class session and reminder emails. The low response rate was possibly due to our efforts ensuring there was no pressure to participate in the study combined with learner fatigue at the end of a 2-h session, as this was a mandatory class activity with optional participation in the research study. Of note, prior to COVID and our expansion to an additional location, we ran similar encounters in-person with the Vital Sign Simulator displayed on the projector and the traditional TBL format of paper questions for the activities and team white-boards for report-outs. The main limitation to this format was physical space for the session, which required us to split the class into two rounds. Because of the distance and physical space limitations, combined with our continued ability to achieve session learning outcomes adequately, we have continued to run the encounter fully remote since COVID.

While our modality was a success, as discussed above, there are areas for improvement. Some students felt overwhelmed. This may be improved by more time on fewer cases, multiple shorter sessions, or earlier distribution of case information. Students occasionally missed patient data given in the group chat during the discussion. During the session, the authors recommend providing all pertinent patient information both in the chat for “real-time” discovery and in the text of the subsequent application exercise for reference. Areas for future research include comparison between TBL and SBLE, direct comparison of SBLE use during various TBL components (e.g., prework, application exercises, discussion), and exploring which SBLE (e.g., Vital Sign Simulator, high fidelity manikin, standardized patients, task trainers, virtual reality, watching recorded simulations, or serious games) are most effective in combination with TBL.

## Conclusion

Using TBL and SBLE together with data-supported learning techniques is endorsed by students and facilitates horizontal and vertical integration. This modality suggests possible improvement in knowledge of essential physiology and pharmacology concepts. In addition, this modality is inexpensive, widely accessible, and adaptable to in-person, hybrid-remote, and fully remote contexts. While demonstrated with pre-health Master of Science in Biomedical Sciences students, it is highly plausible that these outcomes would translate to learners in undergraduate, graduate, doctoral, and professional programs.

## Supplementary Information

Below is the link to the electronic supplementary material.

Supplementary file1 (DOCX 14 KB)

## Data Availability

The datasets generated during and/or analyzed during the current study are available from the corresponding author on reasonable request.
