# Supplementary material for: Integrating Foundational and Clinical Science Remotely by Combining Team-Based Learning and Simulation
Source: Med Sci Educ. 2023 Jun 15;33(4):925–34. doi: 10.1007/s40670-023-01817-9 (PMC10403461; doi:10.1007/s40670-023-01817-9)
Supplement: Supplementary file 1 — Supplementary file1 (DOCX 14 KB) [file 40670_2023_1817_MOESM1_ESM.docx]

MSBS Physio-Vital Sign Simulation

**Pre- and post-5 questions**

1. Acetylcholine constricts the pupil. Which of the following drug causes miosis as well?

A. Atropine

B. Opioids

C. Metoprolol

D. Cocaine

E. Diazepam

2. Adrenal tumors can be located in the cortex and medulla. Which molecule in the blood is most likely increased if it is an adrenal medulla tumor?

A. Nicotine

B. Acetylcholine

C. Metanepherines

D. Serotonin

E. Glutamine

3. A dose of atropine is intravenously injected into a 30 y/o male. Which of the following is most likely to happen after the injection?

A. Miosis

B. Diarrhea

C. Diaphoresis

D. Tachycardia

E. Bronchospasm

4. Epinephrine is the drug of choice to rescue a patient from a severe allergic reaction, which presents with hypotension and bronchospasm. What does epinephrine induce?

A. Decreased blood pressure

B. Decreased heart rate

C. Dilated bronchus

D. Diarrhea

E. Miosis

5. Which reaction is induced by nicotinic receptor activation?

A. Lacrimation

B. Bronchospasm

C. Bradycardia

D. Miosis

E. Skeletal muscle twitch

Correct answers: 1: B; 2: C; 3: D; 4: C; 5: E.
